# Research progress on endovascular treatment of Stanford Type A aortic dissection

**DOI:** 10.3389/fcvm.2025.1641559

**Published:** 2025-09-04

**Authors:** Yue Xiao, Yang Zhang, Heng Li, Yuanyuan Guo

**Affiliations:** Vascular Surgery Department, Fuwai Yunnan Cardiovascular Hospital, Kunming Medical University, Kunming, China

**Keywords:** Type A aortic dissection, aortic root, ascending aorta endovascular repair, endovascular vascular graft, ascending aortic

## Abstract

Thoracic endovascular aortic repair (TEVAR) has become the primary treatment for Stanford Type B aortic dissection. With the development of new endovascular grafts for the aortic arch, endovascular treatment for lesions involving all three branches of the aortic arch will gradually be applied in clinical practice. However, Stanford Type A aortic dissection (TAAD), owing to the anatomical characteristics of the aortic root and ascending aorta segment, as well as hemodynamic features, remains a formidable challenge in endovascular repair of aortic diseases. Endovascular repair of Type A aortic dissections presents several unresolved challenges, including significant anatomical variability (e.g., involvement of the coronary arteries, aortic valve, and diverse dissection morphologies), the ability of novel grafts to conform to complex anatomy, the integration of endovascular stents with valve interventions, and the maintenance of long-term coronary artery patency. Currently, there is no ideal endovascular solution or commercialized graft available. This article reviews the research progress in endovascular treatment for TAAD and outlines future technical development directions.

## 1 Introduction

With the rapid development of parallel stent technology, extracorporeal/*in situ* windowing techniques, and endovascular grafts with single or multiple branches, areas previously considered inaccessible to endovascular repair, such as the three branches of the aortic arch and the visceral artery region of the descending aorta, have been gradually overcome (1, 2). Traditional open surgeries such as Bentall or Wheat procedures (aortic valve and ascending aorta replacement, with or without coronary artery bypass grafting) performed under extracorporeal circulation remain the gold standard for treating lesions involving the aortic root and ascending aorta. Although open surgery has good long-term outcomes, more than 30% of Type A dissection patients are elderly or high-risk, and the mortality rate of traditional open surgery reaches 20% (3, 4). For elderly patients, those at high surgical risk, or those with contraindications for open surgery, endovascular repair as an alternative to open surgery is gaining increasing attention, and the accessibility of endovascular repair far exceeds that of open surgery. How to utilize endovascular techniques to overcome the so-called forbidden zone of the aortic root-ascending aorta and address the final 5 cm of endovascular aortic surgery has become a focal point for leading vascular surgery centers worldwide.

For some Debakey Type II dissections or patients with localized ulcers or aneurysms in the ascending aorta, many medical device manufacturers have developed stents specifically designed for the ascending aorta (5, 6). However, currently available ascending aorta stent grafts all require the proximal anchorage zone to be located at the distal end of the sinus junction (more than 2 cm from the high coronary artery opening), which limits their application to lesions confined to the ascending arch and not involving the aortic root. Clinically, however, the vast majority of TAAD also involve the aortic root. The anatomical characteristics of the aortic root-ascending aorta segment remain a major bottleneck limiting endovascular repair of lesions in this region.

Therefore, this paper reviews and summarizes existing endovascular repair strategies for TAAD from the perspectives of anatomical characteristics, hemodynamic features, challenges in endovascular repair of this region, and current solutions, with the aim of providing a theoretical foundation for the development of novel grafts.

## 2 Anatomical and hemodynamic characteristics of the aortic root and ascending aorta

### 2.1 Anatomical characteristics

The aortic root consists of the left ventricular outflow tract, aortic valve, aortic valve annulus, aortic sinus (coronary artery openings), and sinus tubular junction. The ascending aorta is relatively short, with a median length of 65 mm (interquartile range: 58–74 mm) (7). Attributable to the direct impact of high-velocity pulsatile blood flow from the left ventricle, turbulence occurs during the cardiac cycle, leading to significant changes in diameter. The diameter variation range from the distal end of the coronary arteries to the proximal end of the innominate artery is (17.4% ± 4.8%) to (13.9% ± 3.5%) (8, 9). The rupture site of TAAD is typically located in the ascending aorta, and the dissection often involves the three major branches of the aortic arch. This unique anatomical structure requires that the endograft design needs to balance hemodynamic adaptability (flexibility and anchoring stability) while protecting critical structures (aortic valve, coronary artery openings, sinus bulge, and brachiocephalic trunk opening).

### 2.2 Hemodynamic characteristics

The hemodynamic changes associated with TAAD are complex and progress rapidly, often resulting from ascending aortic aneurysms, aortic sinus aneurysms, and aortic root lesions. Its core features include acute aortic regurgitation, organ perfusion impairment, and the risk of pericardial hemorrhage (10–12). It frequently involves the aortic root, resulting in annular dilation or leaflet prolapse, thereby causing aortic regurgitation. The resulting elevation in left ventricular diastolic pressure and pulmonary edema further exacerbate the condition; organ perfusion impairment depends on the extent of dissection involvement, such as coronary artery involvement leading to acute myocardial infarction or brachiocephalic trunk involvement potentially causing stroke. Moreover, pericardial tamponade is a life-threatening complication of TAAD; once blood enters the pericardial cavity, cardiac diastolic function is impaired, and patients may exhibit Beck's triad, requiring urgent intervention. Given the malignant hemodynamic cycle of it, early surgical intervention is critical.

### 2.3 New subdivision based on the anatomical characteristics of the root-ascending aorta segment

**Figure 1:**
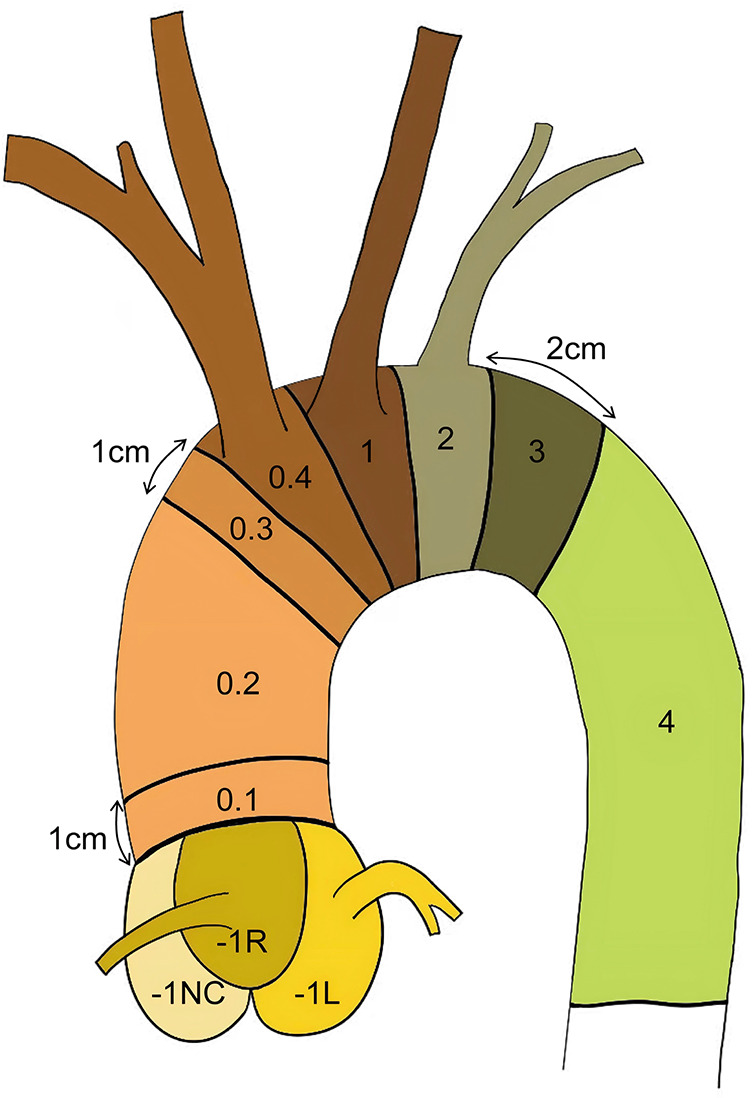
Schematic diagram of aortic segmentation: zone 0.1: The region more than 1 cm away from the sinus junction Zone 0.2: The ascending aorta segment between Zones 0.1 and 0.3 Zone 0.3: The region 1 cm proximal to the origin of the innominate artery Zone 0.4: The aorta segment corresponding to the level of the innominate artery opening Zone −1l: Left coronary sinus (Valsalva sinus).

To meet the growing demands of endovascular treatment for aortic root diseases, Vallée et al. proposed a refined segmentation strategy based on the Ishimaru classification (13), dividing Zone 0 into four segments and introducing a new specific region (Zone-1) at the junction of the sinus cavernosus and the aortic valve annulus. This anatomical classification provides a critical theoretical basis for further research in this field. Its primary purpose is to optimize the development of future treatment strategies and facilitate the comparison of devices. Furthermore, this classification aims to assess the anatomical suitability of using specialized aortic root endovascular grafts for complete endovascular treatment of acute Stanford Type A aortic dissection (ATAAD) and to guide the selection of stent anchorage zones for transcatheter aortic endovascular repair. Nevertheless, the extent of involvement of the root-ascending aorta in Type A dissections varies, and endovascular approaches also vary, necessitating more precise classification to provide clearer endovascular repair strategies (Figure 1).

## 3 Technical challenges of endovascular treatment

### 3.1 Physiological challenges

The aortic root demonstrates complex multidimensional motion characteristics during the cardiac cycle, with axial displacement reaching an average of 7.37 ± 1.96 mm and circumferential rotation angle up to 11.8 ± 4.60° (14, 15). These motion characteristics substantially elevate the risk of graft displacement post-implantation. If larger-sized stents are selected to enhance wall adhesion and anchor stability, excessive mechanical strain may cause vascular damage or even induce new intimal tears (16). Moreover, the distinctive “Windkessel effect” of the ascending aorta plays a critical role in maintaining coronary artery blood flow (17–19). During diastole, the ascending aorta propels stored blood toward distal peripheral branches through elastic recoil while providing retrograde blood flow perfusion to the coronary arteries. Nevertheless, type A dissection lesions result in loss of arterial wall elasticity, markedly diminishing its expansion-recoil capacity. If a stent is placed over the root or ascending aorta, it may restrict local vascular elastic expansion, thereby negatively impacting coronary artery hemodynamics (20, 21).

### 3.2 Technical challenges

Although there have been case reports of endo-bentalld in clinical practice, the grafts used were all customized, differing from endovascular repair of the arch or descending aorta. The technical challenges of the root-ascending aorta segment primarily lie in the following aspects: (1). Anchoring difficulties: The blood flow pressure in the aortic root is high, and the closer to the valve, the stronger the blood flow impact, which may lead to stent displacement (21). (2). Positioning challenges: The most commonly used approach for TEVAR is the femoral artery route, but the graft must navigate multiple “bends” to reach the root-ascending aorta. When endovascular reconstruction of the coronary arteries or percutaneous valve replacement is required, the femoral artery approach may increase positioning difficulties and elevate the risk of stroke. In contrast, the transapical approach has a shorter path but increases the risk of pericardial effusion and ventricular aneurysm (22). (3). Stent applicability issues: The goal of treating aortic dissection is to stabilize the dissection, prevent further tearing, and avoid aortic rupture. However, traditional stents are typically suitable for the subacute phase of the disease, while acute type A dissections present acutely, with the vascular wall in a state of acute edema and tissue fragility, making it difficult to provide a healthy anchoring area (16). 4. Lack of specialized stents: Currently available stent systems require a 20 mm anchoring zone, while the average length from the Sino-Tubular Junction (STJ) to the brachiocephalic trunk is only 70–80 mm. Considering that each end requires a 20 mm anchorage zone, the actual length available to cover the diseased area is reduced to 30–40 mm. This further narrows the indications for stents, limiting their use to cases where the dissection is confined to the ascending aorta. However, TAAD often involves the aortic root or areas near coronary artery openings, making effective occlusion challenging.

### 3.3 Challenges in applying grafts to different anatomical conditions

Clinical data indicate that only 35.9%–46.0% of tears within TAAD are located in the ideal mid-distal segment, while 14.0%–25.2% of primary tears are located in the proximal segment, 15% in the root, and 50%–80% of cases involve the STJ region (13, 23). Therefore, when conducting endovascular graft therapy at the current stage, stents need to be customized according to the patient's individual lesion characteristics, and further development of more comprehensive solutions are required to meet the needs of different morphological conditions. Moreover, the proximal segment of most dissection patients cannot provide sufficient length of normal anchorage area. With regard to the exact required distance, there is currently no consensus, but it is generally believed that the distance from the nearest coronary artery to the entrance tear should be maintained at least between 10 and 20 mm (24, 25). To address this limitation, modern endovascular techniques have developed various solutions such as fenestration, branching, chimney, and bypass. However, it is worth noting that the clinical evidence for endovascular treatment of TAAD primarily comes from case reports and a few small-scale studies. Furthermore, the ascending aorta is a curved structure with an outer curvature significantly longer than the inner curvature, which makes it challenging for traditional stent grafts to fully conform and precisely deploy the stent (26, 27).

The challenges and issues mentioned above constitute only a portion of the difficulties encountered in the development of TAAD-specific stents. In subsequent research and development, numerous details necessitate further optimization, such as the compatibility of stent material compliance with TAAD vascular compliance and changes in hemodynamics following stent implantation.

## 4 Customized stents for the ascending aorta

### 4.1 Cook stent

The Zenith Ascend (Cook Medical) is an endoluminal stent-graft specifically designed for ascending aortic lesions. Introduced in 2015, it incorporates a 10-mm proximal sealing zone and is available in diameters of 28–46 mm with a fixed length of 6.5 cm (28). In 2016, Tsilimparis et al. conducted a multicentre registry including 10 patients treated with the Zenith Ascend and followed for 10 months. Anatomical eligibility required a ≥10 mm landing zone distal to the coronary ostia and proximal to the patent ductus arteriosus, and an aortic diameter of 24–40 mm. The device is indicated in high-risk patients unsuitable for open surgery, for both acute and elective scenarios (29). Approved indications encompass ascending aortic coarctation, saccular aneurysms, and intra-operative stabilization of malpositioned aortic valves. Early outcomes demonstrated 100% technical success and 90% 30-day survival. Major complications comprised endoleak and intimal-tear-related haemorrhage. One patient succumbed to circulatory collapse secondary to persistent type Ia endoleak within 24 h. At a median follow-up of 12 (0–36) months, three late deaths occurred, one attributable to graft infection. These preliminary data support the relative safety and feasibility of the technique in a high-risk cohort (18). In 2019, Tsilimparis reported a single-centre update comprising 24 patients treated with the Zenith Ascend for acute or chronic type A dissection, pseudoaneurysm, and valve malposition. Clinical success was achieved in 90% of cases, whereas 30-day mortality reached 21%. Major adverse events included stroke and endoleak, necessitating secondary interventions in three patients. Although initial results were encouraging, global production has ceased. Study limitations comprise a small, heterogeneous cohort and off-label use, limiting generalisability.

### 4.2 Relay stent

The Relay Custom Medical Device is a patient-specific thoracic stent-graft featuring a dual-fixation delivery platform that enables the proximal portion to conform to the native aortic curvature and to be repositioned intra-operatively. A dedicated non-invasive support wire secures the distal end, thereby preventing collapse or migration. The system enables complete exclusion of the ascending aorta and facilitates treatment of aortic arch lesions via a limited-access approach.Piffaretti et al. retrospectively reviewed nine inoperable patients who underwent total ascending-aortic endoluminal repair with the Relay device for coarctation, pseudoaneurysm, or aneurysm with associated dissection. Procedural success was 100%, with no intra-operative mortality or major complications (e.g., endoleak, conversion to open repair). At a median follow-up of 26 months, three deaths occurred; one resulted from an infected aorto-atrial fistula requiring open revision. Sixty-seven per cent of patients remained asymptomatic (30).

However, the retrospective multicentre design introduced heterogeneity in patient selection, device configuration, and operative technique, and the absence of a contemporaneous open-surgery control cohort may have biased the results. Nonetheless, the study provides proof-of-concept and mid-term data; larger prospective controlled trials are required to enable multifactorial analysis.

### 4.3 Gore stent

The GORE® Ascending Stent Graft is indicated for high-risk patients presenting with acute Stanford type A aortic dissection. The ARISE Early Feasibility Study was a multicentre, prospective, non-randomized, single-arm trial. Nineteen patients were enrolled. Inclusion criteria were: (1) confirmed DeBakey type I or II aortic dissection; (2) entry tear ≥2 cm distal to the most caudal coronary ostium; and (3) true-lumen and total aortic diameters compatible with the 24–42 mm device range. Major complications comprised adverse cardiovascular events, stroke, and endoleak. Within 30 days, five serious adverse cardiovascular events were recorded (all-cause mortality 15.8%), including cerebral infarction in 15.3% and myocardial infarction in 5.3% of patients. Additionally, five patients sustained a stroke and six developed endoleak; four required secondary intervention. Early feasibility data suggest a high technical success rate and an acceptable safety profile for this patient-specific stent-graft in treating Stanford type A dissection. A phase II investigation, initiated in 2023, will expand enrolment to additional centres (31). As the first-in-human evaluation of the GORE® ASG, these findings provide preliminary safety evidence for endoluminal repair of ascending aortic dissection; however, validation through larger, randomized, controlled trials is required given the limited sample size, uncontrolled design, short follow-up, and restrictive enrolment criteria.

## 5 Clinical efficacy of endovascular treatment for TAAD

Since Dorros et al. first described balloon-expandable covered stents for thoracic aortic dissection in 2000, TEVAR has advanced substantially in the management of acute aortic dissection (AAD) (32). Li et al. reported a single-centre series of 15 inoperable patients treated with TEVAR, observing procedure-related complications—including ventricular tachycardia and new-onset coarctation—in 33% of cases; these events were managed successfully with structured follow-up and prompt reintervention (33). Accumulating clinical evidence indicates that TEVAR, as an alternative for high-risk patients, yields significantly superior short-term outcomes compared with conservative therapy, achieving an aorta-related mortality of approximately 5%, particularly in critically ill individuals requiring bridging therapy (21, 22).Two contemporary studies have provided new evidence for TEVAR. A meta-analysis by Lu et al. (*n* = 1,013) reported a zone 0 peri-operative mortality of 7.49%, with major complications of stroke (8.95%) and type Ia endoleak (9.01%); stent migration was the principal driver of endoleak (34). Mylonas et al. (*n* = 311) demonstrated durable mid-term efficacy, with 1- and 5-year survival rates of 87.15% and 82.31%, respectively, and a re-intervention rate of 8.38% (6). These findings underscore the therapeutic utility of TEVAR in high-risk cohorts, yet highlight the need for continued refinement of zone 0 technical protocols and device selection.

Endoleak and stroke remain the most frequent complications after endoluminal therapy. Controversy persists regarding whether endoleaks promote false-lumen enlargement and elevate the risk of re-rupture (35). Post-operative aortic remodelling constitutes a critical surrogate of procedural success. Ghoreishi et al. documented favourable remodelling at a median follow-up of 13 months (36). Furthemore, Li et al. observed sustained favourable remodelling over 71 months, with complete false-lumen thrombosis in all DeBakey type I cases (33).

Current endoluminal series predominantly address DeBakey type II or focal lesions; most stent designs and supporting evidence derive from this subgroup, and further innovation is required to address the anatomical complexity of DeBakey type I dissection.

## 6 Advances in endovascular treatment for Type A aortic dissection

### 6.1 Insights from transcatheter aortic valve implantation (TAVI)

Since Cribier et al. first demonstrated the feasibility of TAVI, the indications for this technique have expanded from high-risk surgical patients to relatively younger patients (under 60 years old) in moderate-to-low-risk populations. However, the long-term durability of interventional valves remains a major challenge (37–39). Recent studies indicate that the lifespan of TAVI valves can match that of surgical biological valves (40). From a technical perspective, the choice between balloon-expandable or self-expanding valves directly influences the overall delivery method of the graft. TAVI is primarily implanted via the femoral artery or transapical approach, and its unique valve-in-valve delivery technique and access selection provide important references for endovascular repair of the aortic root and ascending aorta segments. Notably, achieving seamless integration between ascending aorta covered stents and TAVI valves has become a major challenge in endovascular vascular surgery. This technical breakthrough not only represents the future direction of aortic surgery but will also become the most important research focus in this field over the next 10–20 years.

### 6.2 Endo-wheat

**Figure 2:**
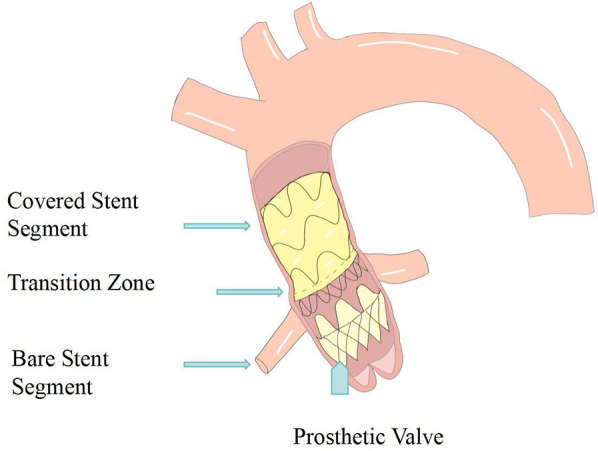
Diagram of the endo-wheat solution.

**Figure 3:**
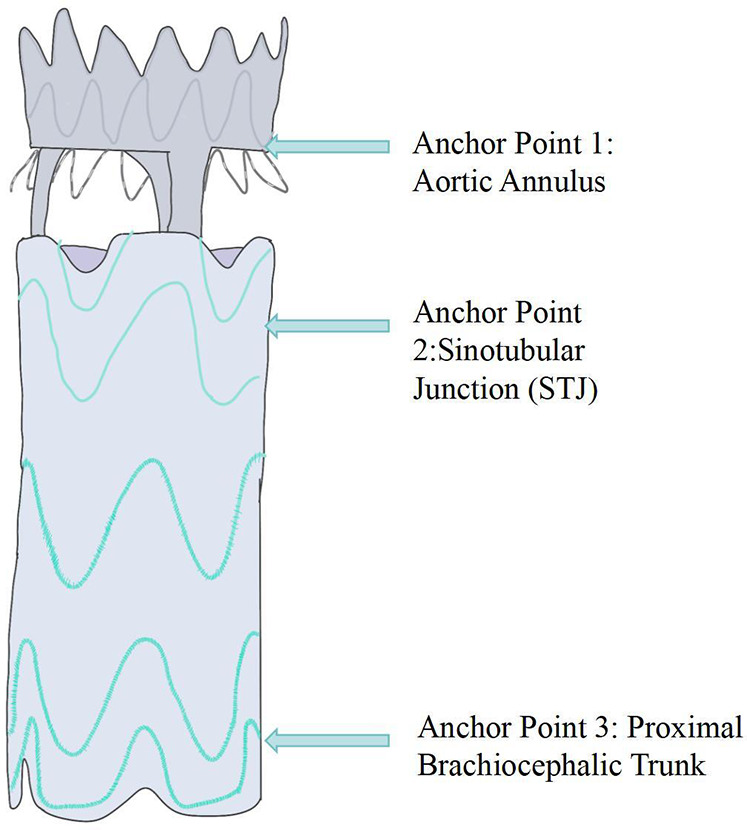
Schematic diagram of the aortic valve component and semi-covered stent graft splice (anchor point 1: aortic Annulus—stabilizes the stent graft via the valve device; anchor point 2: sinotubular junction—forms the proximal sealing zone; anchor point 3: proximal brachiocephalic trunk—forms the distal sealing zone.

In 2014, Rylski et al. proposed an integrated endovascular treatment concept combining TEVAR and TAVI, involving the use of a straight-tube ascending aorta-covered stent graft and an artificial heart valve to treat patients with concurrent ascending aorta and aortic valve diseases during surgery. The composite endograft comprises a proximal transcatheter aortic valve bioprosthesis integrated with a partially covered stent graft, deployable via a transapical approach. To prevent migration, the device incorporates three distinct anchoring zones: the aortic annulus, the sinotubular junction, and the distal ascending aorta. The proximal segment is constructed as a bare stent to preserve unobstructed coronary ostial flow (Figures 2,3).

This prospective cohort enrolled 51 patients: 19 underwent TAVI, 10 underwent open Wheat procedure, and 22 received conservative management. Computed tomography revealed that 41% of patients satisfied the annular diameter criteria for currently approved TAVI prostheses; the novel 30-mm bioprosthesis increased the eligible cohort to 78%. Graft diameters ranged from 30 to 46 mm. All but six patients achieved optimal oversizing (≤10%). In 88% (45/51) of subjects, the minimum annulus-to-coronary-ostium distance was ≥10 mm. Mean distances from the left and right coronary ostia to the sinotubular junction were 2.6 ± 1.5 mm and 3.2 ± 1.7 mm, respectively.

At present, high-risk surgical candidates with severe aortic stenosis and concomitant ascending aortic aneurysm are frequently deemed ineligible for TAVI, because no effective endovascular therapy exists for the dilated ascending aorta. This study proposes an innovative endovascular composite valved graft, it's a novel therapeutic option for this previously untreatable cohort (41).

### 6.3 Endo-bentall

**Figure 4:**
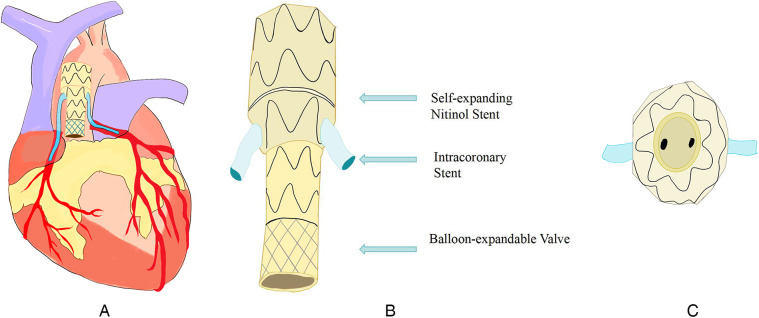
**(A)** Conceptual diagram of the endo-bentall solution; **(B)** schematic diagram illustrating the key component design of the Endo-Bentall solution; **(C)** Top view diagram of the Endo-Bentall solution.

The Endo-Bentall procedure is an innovative endovascular treatment approach that integrates TEVAR, TAVI, and dual coronary stent grafting techniques (Figure 4). This technique is currently primarily used in case reports and small-scale studies (see Table 1). Its clinical application still faces numerous challenges. (1). Anatomical adaptability issues: 40%–50% of TAAD patients experience aortic valve insufficiency due to root deformation. Traditional TAVI devices may shift or cause paravalvular leaks. (2). Technical bottlenecks in coronary artery reconstruction: lack of specialized stents, risk of fracture, and immature coronary artery precise membrane perforation technology. (3). Conflicts in treatment timeliness: Customized stents take a long time to produce, conflicting with the emergency treatment requirements of TAAD.

**Table 1:** Summary of technical applications related to the Endo-Bentall procedure.

Author, date of publication	Patient's age	Type of disease	Access point	Diseased valve	Bracket type	Articulating valve types	Coronary revascularization procedures
Felipe Gaia et al. (42)	64	Pseudoaneurysm of the ascending aorta	Transapical route	Bioprosthetic valve failure	Self-expanding laminating stents	Ball Expandable Valve	Coronary Bridging Stent
Gandet et al. (43)	82	Ascending aortic aneurysm	Transapical route and femoral artery junction channel	Primary aortic stenosis	Physician-modified open-window grafts	Ball Expansion Valve	Prosthetic window
Ghoreish et al. (44)	63; 85	Type A aortic coarctation; ascending aortic aneurysm combined with multiple ruptures	Femoral artery	Severe aortic valve insufficiency; rupture involving the valve	Modified thoracic aortic stent	Self-Expanding Valve	Prosthetic window
Leshnower et al. (45)	71	No coronary sinus-left atrial fistulae	Femoral artery	–	Modified thoracic aortic stent	Ball Expandable Valve	Prosthetic window
Ghoreishi et al. (46)	Average age 79	Four patients with type A aortic coarctation and one with aortic root aneurysm	Femoral artery	Severe aortic valve insufficiency in 2, micro regurgitation of the aortic valve in 1	Modified thoracic aortic stent	Self-Expanding Valve	prosthetic window

In recent years, most literature indicates that approximately two-thirds of patients can undergo treatment with a valve-bearing graft; however, there are currently no commercially available stents specifically designed for TAAD (47, 48).

## 7 National studies in China

When the primary entry tear abuts the brachiocephalic trunk, reconstruction of the three aortic-arch branches is mandatory to secure an adequate proximal sealing zone. Recently, arch-dedicated endografts—including Zipper aortic arch stents, WeFlow-Arch modular inlay, and CS stents—have demonstrated technical feasibility (49–51). Nevertheless, these devices remain investigational and are primarily intended for aortic-arch pathology rather than for TAAD.

In their exploration of endovascular repair of the ascending aorta, Jing Zhaiping's team proposed a classification system based on early clinical data and literature review, dividing the ascending aorta into three regions according to the location of the intimal tear: (1) Distal third: Branched grafts, hybrid surgery, or windowing techniques may be used to preserve head and upper limb perfusion (52–54). (2) Middle third: Tubular covered stents are generally considered appropriate (33, 55). (3) Proximal third: Providing an adequate proximal landing zone remains a challenge (56). The team preliminarily demonstrated the feasibility of endovascular repair for tears in the distal and middle thirds through animal studies and limited clinical observations (median follow-up of 26 months). However, further validation through larger patient cohorts and extended follow-up is necessary.

## 8 Conclusions and outlook

The anatomical, biomechanical, and hemodynamic characteristics of the aortic root and ascending aorta differ substantially from those of the descending thoracic aorta, necessitating further exploration to provide new insights and directions for the development of novel grafts. Current solutions primarily consist of small-sample case reports or series studies from multiple centers, resulting in low levels of clinical evidence (42–46). Future large-scale cohort studies are needed to further validate their safety and efficacy. Innovative endovascular repair of TAAD offers substantial clinical value. First, it avoids cardiopulmonary bypass and thus mitigates the perioperative risks inherent to open surgery, making it particularly suitable for elderly patients or those with multiple comorbidities. Second, it resolves contemporary device limitations regarding coronary ostial preservation and secure proximal fixation. Third, it meets the unmet need for the approximately 30% of patients who are medically ineligible for open repair.

Key future directions in stent graft research include developing specialized stents adapted to the hemodynamics of the ascending aorta, optimizing the design of valve-stent composites, and establishing a standardized anatomical indication assessment system. Additionally, endovascular treatment for ascending aortic dissection remains an off-label application at present, necessitating rigorous assessment of individual patient conditions and cautious selection of treatment options. With the deepening of theoretical research, technological innovations, and the development of new stents, Continued progress in this area may eventually enable the routine application of endovascular techniques in the ascending aorta.

## References

[1] De Freitas S Rossi MJ Abramowitz SD Fatima J Kiguchi MM Vallabhaneni R Systematic review and meta-analysis of endovascular interventions for Stanford type A aortic dissection. J Vasc Surg. (2021) 74:1721–31.e4. 10.1016/j.jvs.2021.01.054 33592292

[2] Harris KM Nienaber CA Peterson MD Woznicki EM Braverman AC Trimarchi S Early mortality in type A acute aortic dissection: insights from the international registry of acute aortic dissection. JAMA Cardiol. (2022) 7:1009–15. 10.1001/jamacardio.2022.2718 36001309 PMC9403853

[3] Hsu ME Chou AH Cheng YT Lee HA Liu KS Chen DY Outcomes of acute aortic dissection surgery in octogenarians. J Am Heart Assoc. (2020) 9:e017147. 10.1161/jaha.120.017147 32912018 PMC7726989

[4] Jiang XL Dong ZH Fu WG. Current status and challenges of endovascular repair for Stanford type A aortic dissection. Zhonghua Wai Ke Za Zhi. (2023) 61:1046–50. 10.3760/cma.j.cn112139-20230921-00138 37932139

[5] Xiao CB Yu HX Mao LF Zhang L Zhang YF Sun KX Surgical treatment of Stanford type A aortic dissection after thoracic endovascular aortic repair. Zhonghua Wai Ke Za Zhi. (2021) 59:520–24. 10.3760/cma.j.cn112139-20200827-00667 34102738

[6] Mylonas KS Zoupas I Tasoudis PT Vitkos E Stavridis GT Avgerinos DV. Endovascular treatment of type A aortic dissection: a systematic review and meta-analysis using reconstructed time-to-event data. J Clin Med. (2023) 12:7051. 10.3390/jcm12227051 38002665 PMC10672308

[7] Li X Zhu L Zhang L Song C Zhang H Xia S Anatomical feasibility study on novel ascending aortic endograft with more proximal landing zone for treatment of type A aortic dissection. Front Cardiovasc Med. (2022) 9:843551. 10.3389/fcvm.2022.843551 35463748 PMC9019117

[8] Van Prehn J Vincken KL Muhs BE Barwegen GK Bartels LW Prokop M Toward endografting of the ascending aorta: insight into dynamics using dynamic cine-CTA. J Endovasc Ther. (2007) 14:551–60. 10.1177/152660280701400418 17696632

[9] Skrypnik D Ante M Meisenbacher K Kronsteiner D Hagedorn M Rengier F Dynamic morphology of the ascending aorta and its implications for proximal landing in thoracic endovascular aortic repair. J Clin Med. (2022) 12:70. 10.3390/jcm12010070 36614871 PMC9821435

[10] Goel NJ Kelly JJ Patrick WL Zhao Y Bavaria JE Ouzounian M Malperfusion in patients with acute type A aortic dissection: a nationwide analysis. Ann Thorac Surg. (2025) 119:980–89. 10.1016/j.athoracsur.2025.01.002 39848556 PMC13095227

[11] Kimura N Aizawa K Kawahito K Itagaki R Yamaguchi A Misawa Y Outcomes of early-onset acute type A aortic dissection- influence of etiologic factors. Circ J. (2019) 83:285–94. 10.1253/circj.CJ-18-0969 30584230

[12] Ji Z Li J Zhong X Sang H Jiang W Zhang H. Comparison of prognoses of patients with type A aortic dissection treated with surgery in acute, subacute and chronic phases. J Thorac Dis. (2025) 17:2227–38. 10.21037/jtd-24-1655 40400933 PMC12090156

[13] Vallée A Guimbretière G Guihaire J Guery A Gaillard M Thomas LH Anatomical feasibility of endobentall strategies for management of acute type A aortic dissection. Ann Surg. (2024). 10.1097/sla.0000000000006548 39351661

[14] Yuan X Kan X Li J Yan Y Mirsadraee S Mittal T Four-dimensional analysis of aortic root motion in normal population using retrospective multiphase computed tomography. Eur Heart J Imaging Methods Pract. (2024) 2:qyae007. 10.1093/ehjimp/qyae007 39045205 PMC11195731

[15] Plonek T Rylski B Nawrocki P Beyersdorf F Jasinski M Kuliczkowski W. Systolic stretching of the ascending aorta. Arch Med Sci. (2021) 17:25–30. 10.5114/aoms.2019.82997 33488852 PMC7811307

[16] Matsumoto T Matsubara Y Aoyagi Y Matsuda D Okadome J Morisaki K Radial force measurement of endovascular stents: influence of stent design and diameter. Vascular. (2016) 24:171–6. 10.1177/1708538115590040 26041700

[17] Iliopoulos DC Kritharis EP Giagini AT Papadodima SA Sokolis DP. Ascending thoracic aortic aneurysms are associated with compositional remodeling and vessel stiffening but not weakening in age-matched subjects. J Thorac Cardiovasc Surg. (2009) 137:101–9. 10.1016/j.jtcvs.2008.07.023 19154911

[18] Atkins AD Reardon MJ Atkins MD. Endovascular management of the ascending aorta: state of the art. Methodist Debakey Cardiovasc J. (2023) 19:29–37. 10.14797/mdcvj.1173 36936356 PMC10022529

[19] Tricarico R Berceli SA Tran-Son-Tay R He Y. Non-invasive estimation of the parameters of a three-element windkessel model of aortic arch arteries in patients undergoing thoracic endovascular aortic repair. Front Bioeng Biotechnol. (2023) 11:1127855. 10.3389/fbioe.2023.1127855 36926690 PMC10011467

[20] Cikach FS Germano E Roselli EE Svensson LG. Ascending aorta mechanics and dimensions in aortopathy—from science to application. Indian J Thoracic Cardiovasc Surg. (2022) 38:7–13. 10.1007/s12055-020-01092-y PMC8980982 35463697

[21] Muetterties CE Menon R Wheatley GH 3rd. A systematic review of primary endovascular repair of the ascending aorta. J Vasc Surg. (2018) 67:332–42. 10.1016/j.jvs.2017.06.099 28844469

[22] Shah A Khoynezhad A. Thoracic endovascular repair for acute type A aortic dissection: operative technique. Ann Cardiothorac Surg. (2016) 5:389–96. 10.21037/acs.2016.07.08 27563553 PMC4973122

[23] Huang C Zhou M Liu Z Huang D Ran F Wang W Computed tomography-based study exploring the feasibility of endovascular treatment of type A aortic dissection in the Chinese population. J Endovasc Ther. (2014) 21:707–13. 10.1583/14-4733mr.1 25290800

[24] Kern M Hauck SR Dachs TM Haider L Stelzmüller ME Ehrlich M Endovascular repair in type A aortic dissection: anatomical candidacy for currently manufactured stent grafts and conceptual valve-carrying devices for an Endo-Bentall procedure. Eur J Cardiothorac Surg. (2023) 63:ezad085. 10.1093/ejcts/ezad085 36916747 PMC10206284

[25] Kreibich M Rylski B Beyersdorf F Siepe M Czerny M. Endo-Bentall for proximal aortic dissection: from conception to application. Asian Cardiovasc Thorac Ann. (2021) 29:697–700. 10.1177/0218492320929211 32436718

[26] Hsu HL Chen CK Chen PL Chen IM Hsu CP Chen CW The impact of bird-beak configuration on aortic remodeling of distal arch pathology after thoracic endovascular aortic repair with the Zenith Pro-Form TX2 thoracic endograft. J Vasc Surg. (2014) 59:80–8. 10.1016/j.jvs.2013.07.098 24139983

[27] Donik Ž Li W Nnate B Pugar JA Nguyen N Milner R A computational study of artery curvature and endograft oversize influence on seal zone behavior in endovascular aortic repair. Comput Biol Med. (2024) 178:108745. 10.1016/j.compbiomed.2024.108745 38901185 PMC11317088

[28] Oderich GS Pochettino A Mendes BC Roeder B Pulido J Gloviczki P. Endovascular repair of saccular ascending aortic aneurysm after orthotopic heart transplantation using an investigational zenith ascend stent-graft. J Endovasc Ther. (2015) 22:650–4. 10.1177/1526602815593537 26112175

[29] Tsilimparis N Debus ES Oderich GS Haulon S Terp KA Roeder B International experience with endovascular therapy of the ascending aorta with a dedicated endograft. J Vasc Surg. (2016) 63:1476–82. 10.1016/j.jvs.2015.12.027 26926935

[30] Piffaretti G Czerny M Riambau V Gottardi R Wolfgruber T Probst C Endovascular repair of ascending aortic diseases with custom-made endografts. Eur J Cardiothorac Surg. (2021) 59:741–49. 10.1093/ejcts/ezaa383 33394032

[31] Roselli EE Atkins MD Brinkman W Coselli J Desai N Estrera A ARISE: first-in-human evaluation of a novel stent graft to treat ascending aortic dissection. J Endovasc Ther. (2023) 30:550–60. 10.1177/15266028221095018 35587698

[32] Dorros G Dorros AM Planton S O'hair D Zayed M. Transseptal guidewire stabilization facilitates stent-graft deployment for persistent proximal ascending aortic dissection. J Endovasc Ther. (2000) 7:506–12. 10.1177/152660280000700612 11194823

[33] Li Z Lu Q Feng R Zhou J Zhao Z Bao J Outcomes of endovascular repair of ascending aortic dissection in patients unsuitable for direct surgical repair. J Am Coll Cardiol. (2016) 68:1944–54. 10.1016/j.jacc.2016.08.031 27788849

[34] Zhu L Li X Lu Q. A systematic review and meta-analysis of thoracic endovascular aortic repair with the proximal landing zone 0. Front Cardiovasc Med. (2023) 10:1034354. 10.3389/fcvm.2023.1034354 36910538 PMC9998709

[35] Sze DY Van Den Bosch MA Dake MD Miller DC Hofmann LV Varghese R Factors portending endoleak formation after thoracic aortic stent-graft repair of complicated aortic dissection. Circ Cardiovasc Interv. (2009) 2:105–12. 10.1161/circinterventions.108.819722 20031703

[36] Ghoreishi M Shah A Jeudy J Pasrija C Lebowitz J Kaczorowski D Endovascular repair of ascending aortic disease in high-risk patients yields favorable outcome. Ann Thorac Surg. (2020) 109:678–85. 10.1016/j.athoracsur.2019.07.015 31472140

[37] Cribier A Eltchaninoff H Tron C. First human transcatheter implantation of an aortic valve prosthesis in a case of severe calcific aortic stenosis. Ann Cardiol Angeiol. (2003) 52:173–5. 10.1016/s0003-3928(03)00062-3 12938570

[38] Kainth AS Sura TA Williams MS Wittgen C Zakhary E Smeds MR. Outcomes after endovascular reintervention for aortic interventions. J Vasc Surg. (2022) 75:877–83.e2. 10.1016/j.jvs.2021.08.090 34592379

[39] Yamashita Y Shimamura K Maeda K Yamada Y Ide T Kuratani T Simultaneous aortic valve-in-valve and ascending stent grafting for prosthetic valve stenosis and ascending flap. Ann Vasc Dis. (2020) 13:422–25. 10.3400/avd.cr.20-00065 33391562 PMC7758572

[40] Rahman F Resar JR. TAVI Beyond 3 years: durability and predictors for survival. Innovations. (2021) 16:417–25. 10.1177/15569845211017558 34182824

[41] Rylski B Szeto WY Bavaria JE Branchetti E Moser W Milewski RK. Development of a single endovascular device for aortic valve replacement and ascending aortic repair. J Card Surg. (2014) 29:371–6. 10.1111/jocs.12348 24762037

[42] Felipe Gaia D Bernal O Castilho E Baeta Neves Duarte Ferreira C Dvir D Simonato M First-in-human endo-bentall procedure for simultaneous treatment of the ascending aorta and aortic valve. JACC Case Rep. (2020) 2:480–85. 10.1016/j.jaccas.2019.11.071 34317269 PMC8311619

[43] Gandet T Westermann D Conradi L Panuccio G Heidemann F Rohlffs F Modular endo-bentall procedure using a “rendez-vous access”. J Endovasc Ther. (2022) 29:711–16. 10.1177/15266028211065959 34964371

[44] Ghoreishi M Chahal D Shah A Kang J Hirsch J Tran D First-in-human endovascular aortic root repair (endo-bentall) for acute type A dissection. Circ Cardiovasc Interv. (2023) 16:e013348. 10.1161/circinterventions.123.013348 37737022

[45] Leshnower BG Duwayri YM Nicholson WJ Ueyama H Gleason PT Shekiladze N Endo-bentall procedure using off-the-shelf catheter devices to repair an aorto-atrial Fistula. Circ Cardiovasc Interv. (2023) 16:e012848. 10.1161/circinterventions.122.012848 37009733

[46] Ghoreishi M Shah A Chahal D Kang J Gupta A Taylor BS Endo-Bentall repair: early results and feasibility of a physician-constructed Endo-Bentall device. J Thorac Cardiovasc Surg. (2025). 10.1016/j.jtcvs.2024.11.041 39824346

[47] Kreibich M Soekeland T Beyersdorf F Bavaria JE Schröfel H Czerny M Anatomic feasibility of an endovascular valve-carrying conduit for the treatment of type A aortic dissection. J Thorac Cardiovasc Surg. (2019) 157:26–34.e1. 10.1016/j.jtcvs.2018.05.045 30041928

[48] Rong D Chen X Han J Yin J Ge Y Liu F Anatomic feasibility of a modular Endo-Bentall stent graft system for type A aortic dissection. J Vasc Surg. (2023) 78:1359–66.e2. 10.1016/j.jvs.2023.07.062 37572892

[49] Dong H Fu W Zhang WW. First-in-man ZIPPER™ endograft system for the treatment of symptomatic aortic arch intramural haematoma. Eur Heart J Case Rep. (2023) 7:ytad574. 10.1093/ehjcr/ytad574 38025117 PMC10681717

[50] Rong D Zhang H Guo W. Aortic arch aneurysm isolated by percutaneous total endovascular arch replacement. Eur Heart J. (2022) 43:2905. 10.1093/eurheartj/ehac326 35706411 PMC9356906

[51] Shu C Wang T Fang K Li Q Luo M He H Concave triple branched stent graft system for aortic arch pathologies: a first in man prospective cohort study. Eur J Vasc Endovasc Surg. (2025). 10.1016/j.ejvs.2025.06.009 40516809

[52] Lu Q Feng J Zhou J Zhao Z Li H Teng Z Endovascular repair by customized branched stent-graft: a promising treatment for chronic aortic dissection involving the arch branches. J Thorac Cardiovasc Surg. (2015) 150:1631–8.e5. 10.1016/j.jtcvs.2015.08.032 26384748

[53] Lin C Lu Q Liao M Guo M Gong J Jing Z. Endovascular repair of the half aortic arch in pigs with an improved, single-branched stent graft system for the brachiocephalic trunk. Vascular. (2011) 19:242–9. 10.1258/vasc.2010.oa0269 21885474

[54] Lin C Wang L Lu Q Li C Jing Z. Endovascular repair of the aortic arch in pigs by improved double-branched stent grafts. Ann R Coll Surg Engl. (2013) 95:134–9. 10.1308/003588413(13511609955814 23484997 PMC4098580

[55] Lu Q Feng J Zhou J Zhao Z Bao J Feng R Endovascular repair of ascending aortic dissection: a novel treatment option for patients judged unfit for direct surgical repair. J Am Coll Cardiol. (2013) 61:1917–24. 10.1016/j.jacc.2012.08.994 23122798

[56] Hagan PG Nienaber CA Isselbacher EM Bruckman D Karavite DJ Russman PL The international registry of acute aortic dissection (IRAD): new insights into an old disease. JAMA. (2000) 283:897–903. 10.1001/jama.283.7.897 10685714

